# The Clinical Manifestation of SARS-CoV-2 in Critically Ill Patients with *Klebsiella pneumoniae* NDM Hospitalized in the ICU of a Modular Hospital during the Third Wave of the Pandemic in Poland—An Observational Cohort Study

**DOI:** 10.3390/diagnostics12051118

**Published:** 2022-04-29

**Authors:** Aneta Guzek, Zbigniew Rybicki, Agnieszka Woźniak-Kosek, Dariusz Tomaszewski

**Affiliations:** 1 Department of Laboratory Diagnostics, Military Institute of Medicine, 04-141 Warsaw, Poland; aguzek@wim.mil.pl (A.G.); awozniak-kosek@wim.mil.pl (A.W.-K.); 2 Department of Anaesthesiology and Intensive Therapy, Military Institute of Medicine, 04-141 Warsaw, Poland; zrybicki@wim.mil.pl; 3 Department of Anaesthesiology and Intensive Therapy, Military Institute of Aviation Medicine, 01-755 Warsaw, Poland

**Keywords:** COVID-19, critically ill, *Klebsiella pneumoniae* NDM

## Abstract

There is limited information on the clinical characteristics of critically ill patients infected with SARS-CoV-2 and *Klebsiella pneumoniae* NDM. The objective of this study was to describe such a group of patients hospitalised in the intensive care unit of a large academic hospital during the third wave of the COVID-19 pandemic in Poland. Between 1 March and 30 June 2021, 103 patients were hospitalised, of whom 23 (22.3%) were positive for *K. pneumoniae* NDM; 14 (61%) of those patients died. Their hospitalisation time varied between 9 and 47 days. Five of the 23 patients (21.7%) were otherwise healthy. In contrast, the others suffered from cardiovascular problems (11, 47.8%), obesity (6, 26.1%), diabetes (5, 21.7%), neurological problems (4, 17.4%), or kidney disease (1, 4.3%); 4 (17.4%) were heavy smokers, and 1 (4.3%) had a history of alcohol abuse. *K. pneumoniae* NDM was isolated from urine samples of all patients. In 17 patients (73.9%), it was also isolated from other sources: from the respiratory tract in 10 (43.8%), from the blood in 2 (8.7%), and the central venous catheter was contaminated in 1 case (4.3%). Fourteen of the patients (60.9%) were colonised *K. pneumoniae* NDM. In four patients (17.4%), bacterial and fungal coinfection occurred. In one case (4.4%), two fungal species, *Candida albicans* and *Candida glabrata*, were isolated simultaneously. The most frequently administered antimicrobial agent was colistin (60.9%), followed by meropenem (47.8%), vancomycin (47.8%), ceftriaxone (34.8%), linezolid (30.4%), piperacillin/tazobactam (30.4%), and trimethoprim/sulfamethoxazole (30.4%). Other less-frequently administered agents included amikacin, amoxicillin/clavulanate, tigecycline, ciprofloxacin, fosfomycin, clindamycin, and cloxacillin. Fluconazole was administered in 14 patients (60.7%) and micafungin was administered in 2 (8.7%).

## 1. Introduction

Until now, more than 300 million patients [1] have been infected with SARS-CoV-2. Many of them have required hospitalisation and critical care. The admission rates to the intensive care unit (ICU) have varied from 5% to 12% in Italy [2,3], from 5% to 26% in China [4,5], and from 5% to 81% in the United States and Canada [6,7,8]. These rates indicate that no less than 15 million patients have been hospitalised in ICUs. Patients hospitalised in the ICU have numerous issues, and the problem of bacterial infections and antimicrobial resistance is also a challenge. Although the relationship between SARS-CoV-2 disease and antimicrobial resistance has not been resolved, some authors [9,10,11,12] suggest such a relation. Regardless of the answer, there is no doubt that any other infection increases the duration and cost of hospitalisation and may worsen the patient’s outcome.

This study aims to describe the characteristics, clinical presentation, antimicrobial treatment, and outcomes of patients with COVID-19 and infected with *Klebsiella pneumoniae* New Delhi metallo-β-lactamase (NDM) hospitalised in the ICU of a large academic hospital during the third wave of the COVID-19 pandemic in Poland.

## 2. Material and Methods

### 2.1. Study Design

This was an observational cohort study. The study design was approved by the institutional Bioethical Committee (37/WIM/2021 of 15 December 2021). Informed concern was waived because no intervention was involved, and no patient identifying information was included.

### 2.2. Setting

This study was performed in an ICU designated for critically ill patients infected with severe acute respiratory syndrome coronavirus 2 (SARS-CoV-2). The ICU was a department of a modular hospital, a part of the Military Institute of Medicine, a 1000-bed academic hospital, and a regional trauma centre. This modular hospital was designed for the treatment of patients infected with SARS-CoV-2. There were 60 beds, including 12 beds in the ICU.

### 2.3. Participants

We analysed the data of all critically ill patients infected with the SARS-CoV-2 hospitalised between 1 March and 30 June 2021, during the third wave of the COVID-19 pandemic in Poland, from whom *K. pneumoniae* NDM had been isolated. No other eligibility criteria were applied.

According to the European Centre for Disease Prevention and Control [13], to perform active surveillance and identify high-risk patients, we performed a swab test (from the respiratory tract or rectal) upon admission of patients to the ICU. Such tests were repeated every seven days of hospitalisation. In every case when the patient had a fever (core temperature ≥38 °C), blood samples were withdrawn and submitted for microbiological testing.

### 2.4. Microbiological Analysis

Bacteria were identified with the VitekMS (bioMérieux, Marcy-l’Étoille, France), an automated microbial identification system that uses matrix-assisted laser desorption/ionization-time of flight (MALDI-ToF) mass spectrometry. Antimicrobial susceptibility was determined for all positive samples except rectal swabs. The broth microdilution method and the automated VITEK-2 system (bioMérieux) were used. The results were read according to the European Committee on Antimicrobial Susceptibility Testing (EUCAST) criteria [14]. The presence and nature of carbapenemase determinants were assessed by molecular testing of bacterial isolates using either a polymerase chain reaction (PCR)-based platform (Xpert-Carba R assay, Cepheid, Sunnyvale, CA, USA) or a lateral flow immunochromatographic assay (RESIST-5 OOKNV, CORIS, BioConcept, Gembloux, Belgium).

### 2.5. Study Size

Due to the retrospective nature of this study, its size was not determined before the start of the project.

### 2.6. Statistical Methods

Collected data were archived and analysed with Microsoft Excel software. Descriptive statistics were used for analysis. The figure was prepared with DataGraph software.

## 3. Results

Between 1 March and 30 June 2021, in the ICU of the modular hospital, 103 patients were hospitalised, including 60 men (58.25%, aged 20–88 years) and 43 women (41.75%, aged 34–97). Twenty-three (22.33%) were positive for multidrug-resistant (MDR) *K. pneumoniae* NDM. All of them were analysed, and no data were missed. Fourteen of 23 patients died (60.87%), and 9 (39.13%) survived. The duration of hospitalisation was between 9 and 47 days (median 22, interquartile range 17–34). The characteristics of the analysed patients, their comorbidities, and empiric and targeted antimicrobial therapy are presented in Table 1. Five of 23 patients (21.74%) were otherwise healthy. Of the 23 patients who were positive for *K. pneumoniae* NDM, 11 (47.83%) suffered from cardiovascular problems, including arterial hypertension (8/23, 34.78%) and heart disease (3/23, 13.04%). Six (26.09%) suffered from obesity, five (21.74%) from diabetes, four (17.39%) had some neurological problems, four (17.39%) were heavy smokers, one (4.35%) suffered from chronic kidney disease, and one (4.35%) had a history of alcohol abuse.

Of the 23 patients positive for *K. pneumonia* NDM, 8 were women (34.78%), and 15 were men (65.22%). The pathogen was isolated from the urine of all patients. In 6 cases (26.09%), this was the only positive sample; in the other 17 patients (73.91%), *K. pneumoniae* NDM was isolated from other sources: from the respiratory tract in 10 (43.48%), from blood samples in 2 (8.70%). The central venous catheter tip was contaminated in 1 case (4.35%). Fourteen of the 23 patients (60.87%) were colonised with *K. pneumoniae* NDM.

Of the 23 patients infected with *K. pneumoniae* NDM, some had superimposed infections. Specifically, 10 cases (43.84%) were positive for MDR *Acinetobacter baumannii*, 2 (8.70%) were positive for *Pseudomonas aeruginosa*, and 1 (4.35%) was positive for methicillin-resistance *Staphylococcus aureus* (MRSA). Moreover, in 4 of 23 patients (17.39%), bacterial and fungal coinfection occurred: in 2 (8.70%) *Candida albicans*, in 1 (4.35%) *Candida glabrata*, and in 1 case (4.35%) both *C. albicans* and *C. glabrata* were isolated simultaneously.

**Figure 1:**
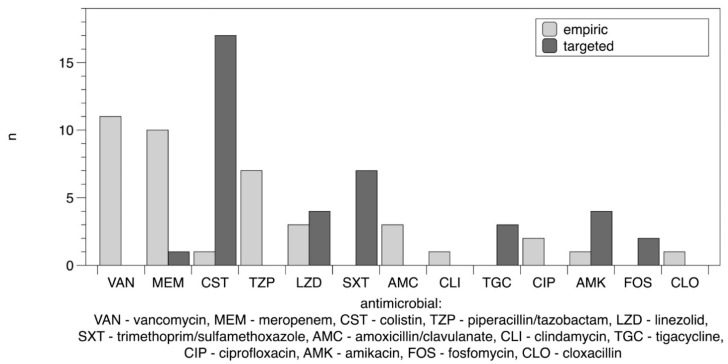
Antimicrobial agents administered in empiric and targeted therapy in critically ill COVID-19 patients hospitalized in ICU infected with *Klebsiella pneumoniae* NDM.

Antimicrobial susceptibility and resistance of *K. pneumoniae* NDM strains isolated from urine samples of all 23 patients were presented in Table 2. The most frequently administered antimicrobial agent was colistin (18/23, 78.26%), followed by meropenem (11/23, 47.83%), vancomycin (11/23, 47.83%), ceftriaxone (8/23, 34.78%), linezolid (7/23, 30.43%), piperacillin/tazobactam (7/23, 30.43%), trimethoprim/sulfamethoxazole (7/23, 30.43%), amikacin (5/23, 21.74%), amoxicillin/clavulanate (3/23, 13.04%), tigecycline (3/23, 13.04%), ciprofloxacin (2/23, 8.70%), fosfomycin (2/23, 8.70%), clindamycin (1/23, 4.35%), and cloxacillin (1/23, 4.35%). Fluconazole was administered in 14 of 23 patients (60.87%), and micafungin was administered in 2 of 23 patients (8.70%). In one case (4.35%), antimicrobial therapy was not administered. The detailed information on the antimicrobials administered in empiric and targeted therapy is shown in Figure 1.

## 4. Discussion

In total, 23 of 103 patients (22.33%) hospitalised in the ICU between 1 March and 30 June 2021, mainly men, had a positive microbiological result for *K. pneumoniae* NDM.

The incidence of carbapenem-resistant *K. pneumoniae* isolation and the gender profile of the patients was similar to the data of Mędrzycka-Dąbrowska et al. [15]. Contrary to their data, in our study, the pathogen was most commonly isolated from urine, not samples from the respiratory tract or the blood.

The incidence of *K. pneumoniae* NDM in our cohort was quite similar to the results of Bentivegna et al. [16], who reported a higher incidence of MDR pathogens in COVID-19 departments compared with other hospital wards (29% vs. 19%), as well as the findings reported by Montrucchio et al. [17]. The high incidence of *K. pneumoniae* NDM may result from complex thoracic pathology related to the COVID-19 infection, mechanical ventilation, exposure to carbapenems and β-lactam/β-lactamase inhibitors, renal replacement therapy, transfusions, and extended inpatient stay [18].

The SARS-CoV-2 infection has a profound impact on the immune system of the infected patients. We still do not know whether coronavirus infection triggers a typical immune response, with cooperation between the natural killer (NK) cells and natural antibodies. However, there is no doubt that the individual immune response is essential in determining the clinical course of SARS-CoV-2 infection [19]. Some parameters were proposed as indicators of individual reactions to the virus and are related to the infection’s clinical course. Hu et al. [20] found a decreased number of lymphocytes, including CD4, CD8, and NK cells, in severe COVID-19 patients. Our study did not analyse the number and changes in such cell lines.

The meta-analysis of Feng et al. [21] found PCT > 0.5 ng/mL, CRP > 10 mg/L a risk factors for disease progression in patients with COVID-19. Moreover, Sayah et al. [22] found that the cut-off value for prediction of mortality is: for IL-6: 83 pg/L (sensitivity 96.3%, specificity 87.6%), for CRP: 15.1 mg/dL (sensitivity 70.4%, specificity 80.0%), for PCT: 0.16 ng/mL (sensitivity 96.3%, specificity 70.5%). In our analysis, of the patients who exceeded these values, 4/7 (57.14%) in the IL-6 group, 14/21 (66.67%) in the PCT group, and 13/19 (68.42%) in the CRP group died. However, a relatively small number of participants makes the comparison of such results pretty difficult.

We administered steroids to all patients to decrease the coronavirus-induced cytokine storm. However, Karruli et al. [23] suggest that MDR infections were more frequent when steroid therapy was administered. It may be one of the reasons for the high incidence of such infections in our cohort.

The information on the relations between the administration of remdesivir and MDR incidence is limited. Nevertheless, it could be pretty challenging to find such a link.

We administered tocilizumab in one case only. Data from Aljuhani et al. [24] revealed the lack of association between such treatment and the incidence of MDR.

The most frequently administered antimicrobial agent in our cohort was colistin, followed by meropenem, vancomycin, ceftriaxone, linezolid, piperacillin/tazobactam trimethoprim/sulfamethoxazole, amikacin, amoxicillin/clavulanate, tigecycline, ciprofloxacin, fosfomycin, clindamycin, and cloxacillin. Comparing such data with the results of other authors is quite tricky, mainly due to the limited data regarding antimicrobial treatment in critically ill patients with COVID-19. In their analysis of 19 studies from China, the United States, Brazil, and Denmark comprising 2834 patients infected with SARS-CoV-2, Chedid et al. [25] found that the primary rate of antibiotic usage was 74%, and the most frequently used antimicrobials were fluoroquinolones, followed by ceftriaxone and azithromycin. Carbapenems were used quite seldomly. One must bear in mind that comparing the whole population of patients infected with SARS-CoV-2 with those who require critical care may produce a critical bias. However, the wide use of antimicrobial agents facilitates the spread of MDR pathogens [26]. The higher incidence of MDR pathogens in patients with COVID-19 compared with the non-COVID-19 population resulting from comprehensive administration of broad-spread antimicrobial agents and selection of resistant pathogens, has been confirmed by Liew et al. [27]. Moreover, the presence of *K. pneumoniae* NDM in the patient room environment, the number of invasive procedures, the inappropriate use of personal protective equipment, and the transmission of pathogens between caregivers and patients may play a role in the aetiology and incidence of such infections [28].

In four of 23 cases (17.4%), we observed both bacterial and fungal infection. These patients died. Such incidence is much higher than noted by Rawson et al. [29], Nori et al. [30], or Hughes et al. [31]. The higher coinfection rate may be the consequence of the patients’ immune state or the fact that all patients analysed in our study received steroids. However, the data reported by Baiou et al. [32] did not support such an observation.

Fourteen of 23 patients positive for *K. pneumoniae* NMD carried this bacterium (60.9%). In recent years, the spread of carbapenemase-producing *K. pneumoniae* has increased in hospitalised patients [33]. However, there are limited data on its incidence in the population of hospitalised patients infected with SARS-CoV-2.

The main limitations of our study are its retrospective design, the lack of clonality data of *K. pneumoniae* NDM isolates, and the fact that it is a single-centre report of only 23 cases; therefore, it is difficult to generalise our results. Moreover, we did not consider the consumption of antimicrobial agents in the study period. Our analysis did not determine how many patients had their position changed to prone. This information could provide some insight because Tiri et al. [10] found that 67% of patients who had changed their posture to a prone position were colonised by carbapenem-resistant Enterobacteriaceae compared with 37% of patients whose posture had not been changed.

## 5. Conclusions

In conclusion, the high incidence of *K. pneumoniae* NDM in our study may result from the clinical profile of hospitalized patients, frequent administration of steroids, and excessive workload of medical personnel, which, due to deficiencies, may lower the standard of sanitary care. Identifying these causes is necessary to reduce the incidence of MDR pathogens and, consequently, improve treatment outcomes.

## Figures and Tables

**Table 1:** The cohort of patients with COVID-19 and isolates causing secondary CPE infections.

Patient Number	Age, Gender	Comorbidities	Duration of Hospitalization (Days)	COVID Treatment	Empiric Antimicrobial Treatment	Targeted Antimicrobial Treatment	Origin of *K. pneumoniae* Isolates	Superimposed Infection	Days from ICU Admission to the Isolation of *K. pneumoniae* NDM in Urine	The Highest Concentration of IL-6	The Highest Concentration of PCT	The Highest Concentration of CRP	Outcome	Occurrence of *Clostridioides difficile*
1	71, M	hypertension Multiple sclerosis	17	remdesivir steroids plasma of convalescents	vancomycin meropenem fluconazole	colistin	urine respiratory tract rectum		7	14.7	0.46	30.1	died	no
2	73, M	diabetes	35	remdesivir steroids clarithromycin	vancomycin meropenem fluconazole	colistin micafungin	urine respiratory tract rectum	*P. aeruginosa* *C. glabrata*	21	NA	0.21	29.4	alive	no
3	46, M	diabetes obesity	9	steroids	vancomycin fluconazole	colistin	urine	*A. baumannii*	2	NA	0.53	23.4	died	no
4	39, M	hypertension obesity depression	14	steroids	ceftriaxone PIP/TAZO	meropenem linezolid fluconazole	urine rectum		14	146.7	4.17	13.3	died	no
5	66, F	hypertension diabetes hypercholesterolemia	23	steroids	vancomycin PIP/TAZO fluconazole	TMT/SMX	urine respiratory tract rectum	*A. baumannii*	9	NA	20.39	67.7	died	no
6	75, F	epilepsy atherosclerosis	30	steroids	amox/clav	colistin	urine respiratory tract		13	NA	0.14	5.0	alive	yes
7	71, M	hypertension alcohol abuse	18	steroids	amox/clav meropenem vancomycin	colistin TMT/SMX	urine		11	NA	8.93	16.5	died	no
8	68, F	CHD	26	remdesivir steroids	ceftriaxone	colistin TMT/SMX linezolid	urine	MRSA *P. aeruginosa*	8	180.7	0.12	14.8	alive	no
9	45, F	obesity hypothyroidism	17	remdesivir steroids	vancomycin meropenem	colistin linezolid fluconazole	urine rectum		5	NA	0.26	32.8	died	no
10	20, M		36	remdesivir steroids	ceftriaxone clindamycin vancomycin	colistin tigecycline linezolid fluconazole micafungin	urine respiratory tract rectum	*A. baumannii* *C. albicans*	4	NA	3.04	27.8	alive	no
11	76, M	CHD heart failure myelodysplastic syndrome history of stroke	34	steroids plasma of convalescents	ceftriaxone ciprofloxacin amox/clav	colistin	urine		30	NA	29.99	21.6	alive	yes
12	74, F	meningioma	16	remdesivir steroids plasma of convalescents	vancomycin meropenem fluconazole	colistin	urine respiratory tract rectum		14	384.8	2.28	41.1	died	no
13	78. M	hypertension	47	steroids	ceftriaxone ciprofloxacin PIP/TAZO	colistin TMT/SMX amikacin fluconazole	urine	*A. baumannii* *C. albicans* *C. glabrata*	5	NA	8.59	48.0	died	yes
14	48, M	hypertension diabetes morbid obesity	29	steroids	ceftriaxone	colistin fosfomycin tigecycline TMT/SMX	blood catheter tip urine respiratory tract	*A. baumannii*	10	NA	46.53	26.0	died	no
15	67, M	CHD hypertension diabetes renal failure	21	steroids	meropenem linezolid	colistin fluconazole	urine rectum	*A. baumannii* *C. albicans*	8	189.1	1.18	30.1	died	no
16	44, M		22	steroids	vancomycin meropenem fluconazole	colistin amikacin	blood urine respiratory tract rectum	*A. baumannii*	3	670.2	30.6	27.6	died	no
17	35, F	obesity	22	steroids	PIP/TAZO linezolid	colistin amikacin TMT/SMX	urine		13	NA	12.71	14.9	alive	no
18	70, M	hypertension	19	remdesivir steroids	vancomycin meropenem fluconazole cloxacillin	*	urine rectum	*A. baumannii*	13	NA	1.7	33.4	died	no
19	77, F	hypertension	18	remdesivir steroids	ceftriaxone	*	urine respiratory tract rectum	*A. baumannii*	12	NA	0.31	27.3	died	no
20	61, M		33	steroids tocilizumab	PIP/TAZO meropenem linezolid	colistin amikacin	urine respiratory tract rectum	*A. baumannii*	6	1331.5	2.83	23.7	alive	no
21	61, M		38	steroids	ceftriaxone amikacin PIP/TAZO	colistin tigecycline fosfomycin fluconazole	urine respiratory tract rectum		22	89.8	0.40	21.8	alive	no
22	43, M	morbid obesity	12	steroids	none	*	urine		6	NA	0.67	15.4	died	no
23	58, M		34	steroids	PIP/TAZO meropenem vancomycin	colistin TMT/SMX fluconazole	urine rectum		25	NA	0.44	31.9	alive	no

CHD—coronary heart disease; CRP—C reactive protein (values in mg/dL); F—female; M—male; MRSA—methicillin resistant Staphylococcus aureus; amox/clav—amoxicillin/clavulanate; IL-6—interleukin 6 (normal range <5.9 pg/mL); PIP/TAZO—piperacillin/tazobactam; PCT—procalcitonin, serum concentration (ng/mL); TMT/SMX—trimethoprim/sulfamethoxazole; * patient died before targeted antimicrobial treatment was administered.

**Table 2:** Antimicrobial susceptibility and resistance of *K. pneumoniae* NDM strains isolated from urine samples of all 23 patients.

Antimicrobial Agent	Susceptibility		Resistance	
	*n*	Percent	*n*	Percent
amikacin (AM)	0	0.0%	23	100%
amoxicillin/clavulanate (AMC)	0	0.0%	23	100%
cefepime (FEP)	0	0.0%	23	100%
cefotaxime (CTX)	0	0.0%	23	100%
ceftazidime (CAZ)	0	0.0%	23	100%
cefuroxime (CF)	0	0.0%	23	100%
cefuroxime axetil (CFA)	0	0.0%	23	100%
ciprofloxacin (CIP)	0	0.0%	23	100%
colistin (CS)	18	78.26%	5	21.74%
fosfomycin (FOS)	12	52.17%	11	47.83%
gentamycin (GN)	2	8.70%	21	91.30%
imipenem (IMI)	0	0.0%	23	100%
meropenem (MEM)	0	0.0%	23	100%
norfloxacin (NOR)	0	0.0%	23	100%
piperacillin/tazobactam (TZP)	0	0.0%	23	100%
tigecycline (TGC)	1	4.35%	22	95.65%
tobramycin (TOB)	0	0.0%	23	100%
trimethoprim/sulfamethoxazole (SXT)	11	47.83%	12	52.17%

## Data Availability

The data presented in this study are available on request from the corresponding author.
